# The long-term effect of job mobility on workers’ mental health: a propensity score analysis

**DOI:** 10.1186/s12889-022-13558-2

**Published:** 2022-06-08

**Authors:** Laura Maniscalco, Martijn Schouteden, Jan Boon, Sofie Vandenbroeck, Ingrid Sivesind Mehlum, Lode Godderis, Domenica Matranga

**Affiliations:** 1 Biomedicine, Neuroscience and Advanced Diagnostics, University of Palermo, Via del Vespro, 129 90127 Palermo, Italy; 2 IDEWE, External Service for Prevention and Protection at Work, Interleuvenlaan 58, 3001 Heverlee, Belgium; 3 Katholieke Universiteit Leuven, Centre for Environment and Health, 3000 Louvain, Belgium; 4 Department of Occupational Medicine and Epidemiology, National Institute of Occupational Health (STAMI), Oslo, Norway; 5 Institute of Health and Society, University of Oslo, Oslo, Norway; 6 Health Promotion, Mother and Child Care, Internal Medicine and Medical Specialties, University of Palermo, Via del Vespro, 133, 90127 Palermo, Italy

**Keywords:** Longitudinal study, Neuropsychological treatment, Depressive disorder, Job mobility, Mental health, Epidemiology

## Abstract

**Objectives:**

The main purpose of this longitudinal study was to elucidate the impact of external job mobility, due to a change of employer, on mental health.

**Methods:**

A cohort of Belgian employees from the IDEWE occupational medicine registry was followed-up for twenty-seven years, from 1993 to 2019. The use of drugs for neuropsychological diseases was considered as an objective indicator of mental health. The covariates were related to demographic, physical, behavioural characteristics, occupational and work-related risks. Propensity scores were calculated with a Cox regression model with time-varying covariates. The PS matching was used to eliminate the systematic differences in subjects’ characteristics and to balance the covariates’ distribution at every time point.

**Results:**

The unmatched sample included 11,246 subjects, with 368 (3.3%) that changed their job during the baseline year and 922 (8.2%) workers that left their employer during the follow-up. More than half of the matched sample were males, were aged less than 38 years old, did not smoke, were physically active, and normal weighted, were not exposed to shift-work, noise, job strain or physical load. A strong association between job mobility and neuropsychological treatment was found in the matched analysis (HR = 2.065, 95%CI = 1.397–3.052, *P*-value < 0.001) and confirmed in the sensitivity analysis (HR of 2.012, 95%CI = 1.359–2.979, *P*-value < 0.001). Furthermore, it was found a protective role of physical activity and a harmful role of job strain on neuropsychological treatment.

**Conclusions:**

Our study found that workers with external job mobility have a doubled risk of treatment with neuropsychological medication, compared to workers without job mobility.

## Introduction

In Europe, between 28 and 33% of the working population has at least one non-communicable disease (NCD), such as diabetes, asthma or depression [1]. NCDs are often the result of a combination of genetic, physiological, environmental and behavioural factors [2]. According to WHO, “mental disorders” belong to the wide class of NCDs and include the broad range of mental and behavioural disorders covered in the F Chapter of the International Statistical Classification of Diseases, tenth revision (ICD-10), such as depression, bipolar affective disorder, schizophrenia, anxiety disorders [2]. About 264 million people suffer from depression or anxiety worldwide and the cost of loss in productivity to the global economy is equal to US$ 1 trillion each year [2]. Mental health is closely connected with work as well as work mobility. Unemployment is associated with poor mental health and psychological distress, and it can have a harmful effect on general health since it is associated with higher mortality rate, hospital admission rate and with long-standing illness [3]. Analogously, working in hostile environment may lead to physical and mental health problems, development of dependence from substances or alcohol, and cause long-term sickness absence and loss of productivity [2]. There is some literature suggesting that job mobility is associated with mental health. In order to give an insight into this relationship, we distinguished between external mobility, defined as changing employer, and internal mobility, defined as changing workplace within the same organization. From now on, we will use the term job mobility referring to external job mobility.

At the worker level, if the job change is voluntary, changing job may have positive effects and lead to improved well-being. In fact, starting a new job is often perceived to improve career advancement and working condition, give increment in salary [4], increase job satisfaction and reduce strain [5–8]. The reasons to decide to change job are usually related to job unsatisfaction, conflicts with supervisors and/or colleagues, high physical or emotional strain, high degree of job insecurity, inadequate working conditions and limited growth opportunities [6, 7]. On the other hand, if the choice to change work does not depend on the worker, as in the case of dismissal or expired employment contract, it is possible that the new job is worse and therefore well-being and satisfaction are reduced. However, the effect of job mobility on health has not been sufficiently investigated.

In the Stanford-Terman longitudinal study [8], higher mortality risk was found in a sample of males experiencing many changes between unrelated jobs, adjusted for education, physical health, anxiety and depression. Regarding cardiovascular outcomes, a Scottish study did not find any association with job mobility [9], while among Belgian workers a change in employment turned out a significant risk factor for being on medication for cardiovascular diseases [10]. Regarding mental health, anxiety and depression were not associated with frequent job mobility in a longitudinal study of Swedish workers [11], while they were associated in a Danish workers population cohort [12]. Regarding obesity, there is evidence from the literature that patients with Major Depressive Disorders show impairment in cognitive domains such as memory, processing speed, and cognitive flexibility [13, 14]. Some recent literature suggests that job strain is a risk factor for mental health and has an important impact on the onset of depression [15, 16]. Regarding shift work, the desynchronization of the circadian rhythms due to night or shift work impacts cognitive performance and tends to increase as shiftwork duration increases, especially for males [17]. Finally, physical activity is recognized as a protective factor, not only for chronic but also for mental illness. Indeed, physical activity is a key factor in the prevention and management of mental health such as depression, stress and anxiety and is useful for mental well-being [18, 19]. The current study aims to assess the relationship between job mobility and mental health in a cohort of Belgian workers followed up for 27 years. Data are drawn from the official registers of the Belgian External Service for Prevention and Protection at Work IDEWE data warehouse and the information regarding the use of a medication for mental health were considered as an objective indicator of mental health disorders. In order to accurately estimate the effect of job mobility on the onset of neuropsychological diseases, a quasi-experimental approach was applied using propensity score matching with time-dependent covariates.

## Materials and methods

### Population and study design

We performed a retrospective longitudinal cohort study of all Belgian worker included in the IDEWE data warehouse, the largest central repository of data on Belgian employees. IDEWE disposes of a database, including data from the annual health surveillance of Belgian employees, recorded and encoded in an electronic format using international or national classification standards [20]. Detailed information about data collection and data warehouse has been described earlier [20, 21]. Periodic health checks in Belgium are mandatory for employees who are exposed to occupational hazards [22]. In addition to medical data, personal and work characteristics are also registered and encoded during medical examination. The data stored in electronic medical files are extracted, translated and loaded into a data warehouse for further analysis.

### Data collection and variables

The dataset includes data on 11,246 employees with measurements in the period between 1993 and 2019, after removing subjects lacking of sex information. The open cohort, with participants entering and leaving the cohort at different times, was followed by the index prescription date from January 1993 to December 2019. The outcome variable was the registry-based information about the use of neuropsychological drugs. It was coded as a binary variable with the “No” category (indicating that a subject in a particular year did not take any medication for neuropsychological diseases) and “Yes”, otherwise. Information on job mobility was provided by the organizations where the respondents were employed. In detail, job mobility was coded equal to “No” if the employee did not change employment or “Yes” if he/she changed employer in a particular year. The covariates included demographic (age, sex), physical and behavioural characteristics (BMI, smoking habits, physical activity), occupational (job mobility) and work-related risks (listed below). A subject was defined physically active if reported working out in line with the WHO recommendations, that is, 30 min of moderate physical activity at least 5 days a week, or at least 20 min a day of vigorous activity for at least 3 days a week, or performed a job or household chores that require important physical effort [23]. However, in the current study, the category related to job or household chores was excluded.

Obesity was dichotomized, considering a cut-off value of 30 for BMI. Number of underweighted (BMI < 18) subjects were negligible in our sample (< 0.1%). Among the work-related risks, the following binary variables were considered: noise, shift-work, manual tasks, job strain, physical load.

Self-reported information about smoking habits, work strain, manual tasks, physical load and shifts work was assessed during the medical examination through the following Yes/No questions: “Are you currently a smoker?”; “Are you currently perceiving work as a strain?”; “Have you been assigned manual tasks?”; “Are you currently perceiving physical load in your work?”; “Are you currently working shifts?”. Only the exposure to noise was measured in dB and the information provided by the employer.

### Statistical analysis

Continuous variables were described as mean and standard deviation (the latter is reported in brackets), median, and range. Categorical variables were analysed as counts and percentages. Quantitative variables were categorized into two classes assuming the median as cut-off value. For all categorical variables, the “No” category was used as a reference. Kuder-Richardson formula 20 was computed to measure inter-items consistency. A high value indicates strong relationship among the items. In order to assess the relationship between covariates and neuropsychological drugs use, first of all the unmatched analysis was performed through the Cox model, as implemented using the “survival” package in the R environment (version 3.5.3). Due to the longitudinal nature of the data and the presence of time-varying variables, the time-dependent data set was built up according to the time-interval format, and the “coxph” function was used to estimate the parameters [24]. Statistically significant variables at univariable analysis were included in multivariable analysis. Afterwards, the same Cox model was applied after propensity score matching. A *p*-value less than 0.05 was considered statistically significant.

### Propensity score analysis

A propensity score analysis was done to balance workers with experience of external mobility (treated group) and workers without this experience (untreated group) to adjust for systematic differences occurring in covariates that are linked to the outcome [25, 26]. The propensity score approach is a quasi-experimental technique widely used in the field of observational studies to mimic randomization of clinical trials [27]. It is used to avoid bias and balance the distribution of the covariates at every time point [27]. The major strengths of propensity score analysis is that it solves the imbalance in covariates between the treated and untreated so that those subjects that cannot be matched are discarded, implying increment of the internal validity and improvement of the quality of observational research, against a reduction of the sample size.

In the Cox model, work mobility was the outcome variable, age and sex were included as fixed covariates, while smoking habit, obesity, physical activity, shift work, noise, manual tasks, job strain and physical load were included as time-dependent variables. The sequential matching algorithm was performed for each risk set in chronological order [27] and the optimal bipartite matching with “optmatch” package was used [28]. Cases were matched with controls with the ratio 1:3 using the hazard of being treated (in our case, the hazard the subjects had to experiment job mobility) at a certain time point for each subject. The selected controls were chosen based on a similar cumulative hazard to the treated in each risk set and matched subjects were removed from the later risk sets. The balance diagnostic of the matched sample was assessed through the standardized mean differences (SMD). Using the strong criterion suggested by Austin [29], we defined a balanced covariate if SMD < 0.1. In order to assess the amount of unmeasured confounders that was not adjusted through propensity score method, we computed the E-value. Whether the E-value is high or low is relative to the magnitude of other covariates’ effect in the study. As an example, if most of the effects have on average a hazard ratio between 1 and 1.5, an E-value equal to 2 is large but, if it is equal to 1.2, it is not. Therefore, the unmeasured confounding should have a relative risk ratio, with both the outcome and the treatment variable (job mobility), at least equal to the E-value to subvert the observed results [30]. The E-value was computed using “EValue” R package [31]. In order to assess the robustness of the association between treatment for neuropsychological disease and job mobility, a sensitivity analysis was made by omitting different matching variables with SMD greater than 0.05 in the unmatched sample.

## Results

The median age of the sample at baseline was 38 years (IQR = 35–51). The unmatched sample included a total of 11,246 subjects, with 368 (3.3%) that changed their job at the baseline (Table 1) and 922 (8.2%) workers that left their employer during the follow-up (data not shown). Age, obesity, and manual tasks showed unbalance between workers with external mobility and workers without. In detail, at baseline, workers aged less than 38 years old were 75.8% with job mobility compared to 46.7% without job mobility. Similarly for obesity, 13% of obese workers changed job compared to 17.2% who did not change job. Furthermore, workers with manual tasks were 79.6% with job mobility compared to 73.2% without job mobility.

**Table 1:** Characteristics of workers stratified by job mobility before (at baseline) and after Propensity Score Matching adjustment

	Unmatched	Unmatched	Matched	Matched	Unmatched	Matched
	No Job mobility	Job mobility	No Job mobility	Job mobility	SMD_u_	SMD_m_
	*n* = 10,878	*n* = 368	*n* = 2319	*n* = 773		
Age (%)						
< 38	5084 (46.7)	279 (75.8)	1628 (70.2)	544 (70.4)	0.625	0.004
≥ 38	5794 (53.3)	89 (24.2)	691 (29.8)	229 (29.6)		
Sex (%)						
Female	4664 (42.9)	167 (45.4)	915 (39.5)	307 (39.7)	0.050	0.005
Male	6214 (57.1)	201 (54.6)	1404 (60.5)	466 (60.3)		
Smoker (%)						
No smoker	7862 (72.3)	269 (73.1)	1726 (74.4)	564 (73.0)	0.018	0.033
Smoker	3016 (27.7)	99 (26.9)	593 (25.6)	209 (27.0)		
Obesity (%)						
No	9012 (82.8)	320 (87.0)	1903 (82.1)	630 (81.5)	0.115	0.015
Yes	1866 (17.2)	48 (13.0)	416 (17.9)	143 (18.5)		
Physical activity (%)						
No	3497 (32.1)	119 (32.3)	692 (29.8)	253 (32.7)	0.004	0.062
Yes	7381 (67.9)	249 (67.7)	1627 (70.2)	520 (67.3)		
Shift work (%)						
No	9062 (83.3)	309 (84.0)	1856 (80.0)	646 (83.6)	0.018	0.092
Yes	1816 (16.7)	59 (16.0)	463 (20.0)	127 (16.4)		
Noise (%)						
No	6922 (63.6)	250 (67.9)	1361 (58.7)	491 (63.5)	0.091	0.099
Yes	3956 (36.4)	118 (32.1)	958 (41.3)	282 (36.5)		
Manual.tasks (%)						
No	2910 (26.8)	75 (20.4)	538 (23.2)	189 (24.5)	0.151	0.029
Yes	7968 (73.2)	293 (79.6)	1781 (76.8)	584 (75.5)		
Job strain (%)						
No	10,857 (99.8)	368 (100.0)	2267 (97.8)	763 (98.7)	0.062	0.072
Yes	21 (0.2)	0 (0.0)	52 (2.2)	10 (1.3)		
Physical load (%)						
No	10,254 (94.3)	342 (92.9)	2030 (87.5)	680 (88.0)	0.054	0.013
Yes	624 (5.7)	26 (7.1)	289 (12.5)	93 (12.0)		

*SMD* Standardized Mean Difference, *SMD*_*u*_ SMD unmatched, *SMD*_*m*_ SMD matched

After PS matching, the matched sample of 3,092 workers had better between-group balancing for all considered characteristics, with SMD < 0.1 for all the covariates (Table 1). More than half of the matched sample included male workers (60.3% in the job mobility group and 60.5% in subjects that did not change job), aged less than 38 years (70.4% in the job mobility group and 70.2% in the no job mobility group), non-smokers (73% in the job mobility group and 74.4% in the no job mobility group), normal weighted (81.5% among subjects who changed job and 82.1% in subject who did not change job), and physically active (67.3% in the job mobility group and 70.2 in the group without job mobility). Furthermore, most of these workers were not exposed to shift-work (83.6% of the job mobility group and 80% of the group without job mobility), noise (63.5% in the job mobility group and 58.7% in the no job mobility group), job strain (98.7% in the job mobility group and 97.8% in the no job mobility group) and physical load (88% in the job mobility group and 87.5% in the no job mobility group) but they usually did manual tasks (75.5% in the job mobility group and 76.8% in the no job mobility group) (Table 1). The Kuder-Richardson formula 20 was equal to 0.84, and suggested inter-items consistency.

In the unmatched sample, job mobility was found a significant risk factor for neuropsychological treatment (HR = 1.330, 95%CI = 1.135–1.559) adjusted for the covariates. Furthermore, all the other covariates showed a statistically significant association with neuropsychological treatment, except for obesity. In the matched sample, job mobility (HR = 2.065, 95%CI = 1.397–3.052, *P*-value < 0.001) was confirmed as statistically significant. Of other covariates, only physical activity (HR = 0.493, 95%CI = 0.332–0.733, *P*-value < 0.001), and job strain (HR = 3.986, 95%CI = 1.593–9.971, *P*-value = 0.003) were statistically significant (Table 2).

**Table 2:** Comparison of hazard ratios for neuropsychological treatment obtained through Cox regression model with time-dependent covariates before and after Propensity Score Matching

	Unmatched				Matched 1:3			
	HR	SE	*P*-value	95%CI	HR	SE	*P*-value	95%CI
Age								
≥ 38 vs < 38	1.290	0.042	** < 0.001**	(1.187—1.403)	1.120	0.249	0.648	(0.687—1.826)
Sex								
Male vs Female	0.474	0.052	** < 0.001**	(0.428—0.526)	0.700	0.238	0.136	(0.438—1.119)
Smoker								
Yes vs No	1.345	0.045	** < 0.001**	(1.230—1.471)	1.226	0.212	0.335	(0.809—1.860)
Obesity								
Yes vs No	1.073	0.051	0.167	(0.970—1.186)	1.116	0.243	0.649	(0.693—1.798)
Physical activity								
Yes vs No	0.575	0.043	** < 0.001**	(0.528—0.627)	0.493	0.202	** < 0.001**	(0.332 -0.733)
Shift work								
Yes vs No	1.161	0.050	**0.002**	(1.053—1.281)	1.192	0.244	0.471	(0.738—1.924)
Noise								
Yes vs No	1.138	0.057	**0.024**	(1.016—1.274)	0.839	0.266	0.511	(0.497—1.415)
Manual tasks								
Yes vs No	1.143	0.056	**0.017**	(1.023—1.278)	1.314	0.278	0.326	(0.761—2.267)
Job strain								
Yes vs No	1.564	0.131	** < 0.001**	(1.209—2.025)	3.986	0.467	**0.003**	(1.593—9.971)
Physical load								
Yes vs No	1.190	0.068	**0.011**	(1.040—1.362)	1.222	0.338	0.553	(0.629—2.373)
Job mobility								
Yes vs No	1.330	0.080	** < 0.001**	(1.135—1.559)	2.065	0.199	** < 0.001**	(1.397—3.052)

*HR* Hazard Ratio, *SE* Standard Error, *95%CI* 95% Confidence Interval

The E-value of treatment for neuropsychological disease was equal to 2.86, and the lower CI limit was 1.99. Based on the magnitude of the other HRs, all but one are less than 1.4, this E-value can be judged as relatively large. The unmeasured confounding should have a relative risk association at least as large as 2.86 with both treatment for neuropsychological disease and job mobility to subvert the results. In the sensitivity analysis to further assess the robustness of the associations, we removed job strain (SMD_u_ = 0.062), obesity (SMD_u_ = 0.115) and manual tasks (SMD_u_ = 0.151) from matching variables. The results after the sensitivity analysis remained consistent and statistically significant, with an HR of 2.012 (95%CI = 1.359–2.979, *P*-value < 0.001).

## Discussion

Our study demonstrated the negative impact of external job mobility on mental health of Belgian workers, as measured through the objective indicator of drugs use for neuropsychological diseases. Our paper’s contribution is noteworthy, since the amount of literature concerning the relationship between job mobility and mental health is very limited, while most of the studies consider burnout, self-reported measures of job satisfaction and work conditions as health outcomes. To the best of our knowledge, only two studies analyse mental health as the outcome, and their findings are not consistent. Our results are consistent with the registry-based longitudinal Danish study of Hoougard that found an adverse effect for both male and female workers [12]. Conversely, another study found no association between job mobility and mental health, but its study target, made of Swedish civil servants, was completely different from our study target [11].

To explain this important result of our study, we can hypothesise health worsening as a consequence of external job mobility-induced stress. The application of the propensity score matching with time-dependent covariates [27], through the balance of the distribution of the covariates, managed to mimic randomization. Successively, the sensitivity analysis assessed the robustness of the strength of the association between treatment for neuropsychological disease and job mobility. The most important advantage of this quasi-experimental approach was the assessment of pseudo-causal effects and not of simple associations.

Current literature showed that the relationship between job mobility and health is bidirectional, pending on contextual characteristics of the work and social environment of the employee. In labor markets with high unemployment and precarious temporary jobs, mobility is often involuntary and between more unhealthy jobs [12]. It is more frequent to observe upward and voluntary mobility with effects on better mental health among high-skilled and high-educated workers [32]. In contrast, if people perceive a gap between their intention to move and the actual possibility of changing jobs, then this effect on health may be negligible or negative. The relationship between job mobility and mental health is confused by several context-related risk factors as well as gender, age and level of education [12]. The method we used made it possible to neutralize the effect of many confounders.

According to psychological theory, any life change, whether perceived as positive or negative, can induce social readjustment and, consequently, stress reaction and arise somatic and mental disorders [12, 33]. Therefore, both voluntary and involuntary mobility can activate such a causal sequence that worsens mental health. Furthermore, both job control and reward at work are important stress conditions that may have an impact on long-term effects on workers’ health [34]. So that, if job change improves the balance between effort and reward, as happens in voluntary and vertical mobility, the health status will improve. On the contrary, it will worsen with the possibility of developing depression [15, 35]. Therefore, having found a worsening impact on mental health due to the job change can be ascribed to involuntary horizontal mobility.

Concerning other results, our study found that job strain is a significant risk factor for mental health while being physically active has a protective role. The harmful role of job strain on the development of neuropsychological diseases is consistent with the results of a longitudinal study conducted on the Canadian population where it was found as the major risk factor of depressive episodes [36] and in a cohort of about one-hundred of full-time workers in Baltimore followed for three years [37]. A multicohort study, together with some meta-analysis including longitudinal studies, showed a prospective association between the increment in job strain and poorer mental health, besides coronary heart disease, stroke and diabetes [15, 16, 38]. Furthermore, the evidence of the protective role of being physically active is consistent with an Italian survey [39] and a systematic review, where the authors demonstrated that aerobic exercise is associated with better psychological health [40].

The main strengths of this paper are the availability of extensive longitudinal data that flow from a twenty-seven-year follow-up study and the use of an objective measure of mental health. In fact, the health status was not self-reported but the use of neuropsychological drugs was retrieved from the IDEWE data warehouse. The third important strength of our study relies on the use of the propensity score matching to create a quasi-experimental context. However, the efficacy of this approach could have been limited by the lack of other important information as work satisfaction, sickness absence, family life, supportive relationships with colleagues, economic security, educational level, and access to social support, healthy behaviours, job control, and workplace characteristics. Furthermore, the lack of information about the distinction between voluntary and forced job mobility and about the specific causes of job mobility cannot exclude the occurrence of other unmeasured confounding in the analysis.

Finally, the healthy-worker effect might have influenced the outcome due to the selection of workers in the labour force and without mental impairment during the twenty-seven years of follow-up. This healthy-worker effect can have underestimated the effects of job mobility. The drop-out of employees that leave their job or change it, with the effect to be lost to follow up, because they are no longer enrolled in the same OSH provider (IDEWE). Moreover, the specific causes of job changes are not considered, so the occurrence of some confounding in the analysis cannot be excluded. Furthermore, self-reported information on smoking habits was potentially underreported.

There are some questions which remain unanswered. For this reason, in future research, we intend to design an ad-hoc study to detect the effect of job mobility in some segments of the working population such as manual vs non-manual, high vs low skilled, and to examine the effect of environmental or chemical exposures to the likelihood of going towards job mobility.

## Conclusion

The main finding of our study was that external job mobility has an impact on mental health. Programs and policies are needed to overcome the negative impact of external job mobility on mental health. Specifically, policies to support workers subjected to voluntary job change should include flexible working hours, exercise, providing competitive salaries, incentivizing workers with rewards and positive reinforcement, and implementing open communication with colleagues and supervisors. Alternatively, workers under involuntary job change should be supported through welfare interventions, professional requalification, and return-to-work programmes. Therefore, it is desirable promoting policies at micro (employer) and macro (government) level to limit the impact of change of work on the mental health of workers.

## Data Availability

The data that support the findings of this study are available from IDEWE but restrictions apply to the availability of these data, which were used under license for the current study, and so are not publicly available. Data are however available from the authors upon reasonable request and with permission of Professor Lode Godderis.

## References

[1] Detaille SI, Heerkens YF, Engels JA, van der Gulden JWJ, Van Dijk FJH (2013). Effect evaluation of a self-management program for Dutch workers with a chronic somatic disease: a randomized controlled trial. J Occup Rehabil.

[2] World Health Organisation. Mental health action plan 2013–2020. 2013. Available from: http://apps.who.int/iris/bitstream/10665/89966/1/9789241506021_eng.pdf

[3] Goodman N. The impact of employment on the health status and health care costs of working-age people with disabilities. LEAD Cent Policy Br. 2015. https://leadcenter.org/wp-content/uploads/2021/07/impact_of_employment_health_status_health_care_costs_0.pdf.

[4] Topel RH, Ward MP (1992). Job mobility and the careers of young men. Q J Econ.

[5] Bernstrøm VH (2013). The relationship between three stages of job change and long-term sickness absence. Soc Sci Med.

[6] De Lange AH, De Witte H, Notelaers G (2008). Should I stay or should I go? Examining longitudinal relations among job resources and work engagement for stayers versus movers. Work Stress.

[7] Swaen GMH, Kant IJ, van Amelsvoort LGPM, Beurskens AJHM (2002). Job mobility, its determinants, and its effects: longitudinal data from the Maastricht cohort study. J Occup Health Psychol.

[8] Metcalfe C, Smith GD, Sterne JAC, Heslop P, Macleod J, Hart C (2003). Frequent job change and associated health. Soc Sci Med.

[9] Cherry N (1976). Persistent job changing—Is it a problem?. J Occup Psychol.

[10] Maniscalco L, Schouteden M, Boon J, Matranga D, Godderis L (2020). The impact of a change in employment on three work-related diseases: a retrospective longitudinal study of 10,530 belgian employees. Int J Environ Res Public Health.

[11] Liljegren M, Ekberg K (2008). The longitudinal relationship between job mobility, perceived organizational justice, and health. BMC Public Health.

[12] Hougaard CØ, Nygaard E, Holm AL, Thielen K, Diderichsen F (2017). Job mobility and health in the Danish workforce. Scand J Public Health.

[13] Rock PL, Roiser JP, Riedel WJ, Blackwell A (2014). Cognitive impairment in depression: a systematic review and meta-analysis. Psychol Med.

[14] Restivo MR, McKinnon MC, Frey BN, Hall GB, Syed W, Taylor VH (2017). The impact of obesity on neuropsychological functioning in adults with and without major depressive disorder. PLoS One.

[15] Aronsson G, Theorell T, Grape T, Hammarström A, Hogstedt C, Marteinsdottir I (2017). A systematic review including meta-analysis of work environment and burnout symptoms. BMC Public Health.

[16] Netterstrøm B, Conrad N, Bech P, Fink P, Olsen O, Rugulies R (2008). The relation between work-related psychosocial factors and the development of depression. Epidemiol Rev.

[17] Rouch I, Wild P, Ansiau D, Marquié J-C (2005). Shiftwork experience, age and cognitive performance. Ergonomics.

[18] Teychenne M, White RL, Richards J, Schuch FB, Rosenbaum S, Bennie JA (2020). Do we need physical activity guidelines for mental health: what does the evidence tell us?. Ment Health Phys Act.

[19] Marques A, Peralta M, Martins J, Catunda R, de Matos MG, Nunes LS (2016). Associations between physical activity and self-rated wellbeing in European adults: a population-based, cross-sectional study. Prev Med (Baltim).

[20] Godderis L, Mylle G, Coene M, Verbeek C, Viaene B, Bulterys S (2015). Data warehouse for detection of occupational diseases in OHS data. Occup Med (Chic Ill).

[21] Bosson-rieutort D, Schouteden M, Godderis L. Observational Surveillance Approach to Detect Novel An Application to a Belgian Occupational Health and Safety Database. 2018 ;60(9):e476–83. 10.1097/JOM.0000000000001399. 10.1097/JOM.0000000000001399 30199412

[22] Godderis L, Johannik K, Mylle G, Bulterys S, Moens G (2014). Epidemiological and performance indicators for occupational health services: a feasibility study in Belgium. BMC Health Serv Res.

[23] Matranga D, Tabacchi G, Cangialosi D (2018). Sedentariness and weight status related to SES and family characteristics in Italian adults: exploring geographic variability through multilevel models. Scand J Public Health.

[24] Therneau T, Crowson C, Atkinson E. Using time dependent covariates and time dependent coefficients in the cox model. Red. 2013;2(1). https://cran.pau.edu.tr/web/packages/survival/vignettes/timedep.pdf.

[25] Rosenbaum PR, Rubin DB (1983). The central role of the propensity score in observational studies for causal effects. Biometrika.

[26] Zhao Q-Y, Luo J-C, Su Y, Zhang Y-J, Tu G-W, Luo Z (2021). Propensity score matching with R: conventional methods and new features. Ann Transl Med.

[27] Lu B (2005). Propensity score matching with time-dependent covariates. Biometrics.

[28] Hansen BB, Fredrickson M. Package ‘optmatch.’ Available https//cran r-project org/web/packages/optmatch/optmatch pdf. Last accessed 10 Oct 2015. 2016

[29] Austin PC (2011). An introduction to propensity score methods for reducing the effects of confounding in observational studies. Multivariate Behav Res.

[30] Haneuse S, Van Der Weele TJ, Arterburn D (2019). Using the E-value to assess the potential effect of unmeasured confounding in observational studies. JAMA.

[31] Mathur MB, Ding P, Riddell CA, VanderWeele TJ (2018). Website and R package for computing E-values. Epidemiology.

[32] Holland P, Burström B, Whitehead M, Diderichsen F, Dahl E, Barr B (2011). How do macro-level contexts and policies affect the employment chances of chronically ill and disabled people? Part I: The impact of recession and deindustrialization. Int J Heal Serv.

[33] Holmes TH, Rahe RH (1967). The social readjustment rating scale. J Psychosom Res..

[34] Siegrist J (1996). Adverse health effects of high-effort/low-reward conditions. J Occup Health Psychol.

[35] Rugulies R, Aust B, Madsen IEH (2017). Effort-reward imbalance at work and risk of depressive disorders. A systematic review and meta-analysis of prospective cohort studies. Scand J Work Environ Health.

[36] Wang J (2005). Work stress as a risk factor for major depressive episode (s). Psychol Med.

[37] Stansfeld S, Candy B (2006). Psychosocial work environment and mental health—a meta-analytic review. Scand J Work Environ Health..

[38] Hanson LLM, Rod NH, Vahtera J, Peristera P, Pentti J, Rugulies R (2019). Multicohort study of change in job strain, poor mental health and incident cardiometabolic disease. Occup Environ Med.

[39] Matranga D, Restivo V, Maniscalco L, Bono F, Pizzo G, Lanza G (2020). Lifestyle medicine and psychological well-being toward health promotion: a cross-sectional study on Palermo (Southern Italy) undergraduates. Int J Environ Res Public Health.

[40] Michie S, Williams S (2003). Reducing work related psychological ill health and sickness absence: a systematic literature review. Occup Environ Med.

